# Development and usability of a web-based patient-tailored tool to support adherence to urate-lowering therapy in gout

**DOI:** 10.1186/s12911-022-01833-6

**Published:** 2022-04-07

**Authors:** Ritch te Kampe, Annelies Boonen, Tim L. Jansen, Jan Mathis Elling, Marcel Flendrie, Yvonne van Eijk-Hustings, Matthijs Janssen, Caroline van Durme, Hein de Vries

**Affiliations:** 1 Division of Rheumatology, Department of Internal Medicine, Maastricht University Medical Center, Maastricht, The Netherlands; 2 Care and Public Health Research Institute (CAPHRI), Maastricht University, Maastricht, The Netherlands; 3 Department of Rheumatology, VieCuri Medical Center, Venlo, The Netherlands; 4 Department of Health Promotion, Maastricht University, Maastricht, The Netherlands; 5 Department of Rheumatology, Sint Maartenskliniek, Nijmegen, The Netherlands; 6 Department of Clinical Epidemiology and Medical Technology Assessment, Maastricht University Medical Center, Maastricht, The Netherlands; 7 Centre Hospitalier Chrétien, Liège, Belgium

**Keywords:** Gout, Medication adherence, Program evaluation, Health information system, Self-management

## Abstract

**Background:**

The aim of this study is to develop and assess usability of a web-based patient-tailored tool to support adherence to urate-lowering therapy (ULT) among gout patients in a clinical setting.

**Methods:**

The content of the tool was based on the Integrated Change (I-Change) model. This model combines various socio-cognitive theories and assumes behavioral change is a result of becoming aware of the necessity of change by integrating pre-motivational, motivational, and post-motivational factors. An expert group (five gout experts, three health services researchers, and one health behavior expert) was assembled that decided in three meetings on the tool’s specific content (assessments and personalized feedback) using information from preparatory qualitative studies and literature reviews. Usability was tested by a think aloud approach and validated usability questionnaires.

**Results:**

The I-Change Gout tool contains three consecutive sessions comprising 80 questions, 66 tailored textual feedback messages, and 40 tailored animated videos. Navigation through the sessions was determined by the patients’ intention to adapt suboptimal ULT adherence. After the sessions, patients receive an overview of the personalized advices and plans to support ULT adherence. Usability testing among 20 gout patients that (ever) used ULT and seven healthcare professionals revealed an overall score for the tool of 8.4 ± 0.9 and 7.7 ± 1.0 (scale 1–10). Furthermore, participants reported a high intention to use and/or recommend the tool to others. Participants identified some issues for further improvement (e.g. redundant questions, technical issues, and text readability). If relevant, these were subsequently implemented in the I-Change Gout tool, to allow further testing among the following participants.

**Conclusion:**

This study provides initial support for the usability by patients and healthcare professionals of the I-Change Gout tool to support ULT adherence behavior.

**Supplementary Information:**

The online version contains supplementary material available at 10.1186/s12911-022-01833-6.

## Background

Gout is the most common type of inflammatory arthritis worldwide [1, 2]. The prevalence and incidence of gout vary widely according to the population studied and methods employed, but range from a prevalence of < 1% to 6.8% and an incidence of 0.58 to 2.89 per 1,000 person-years [2]. An elevated serum uric acid (sUA) is the main risk factor for gout. Both lifestyle and comorbidities contribute to hyperuricemia and possibly independently also to gout. Fortunately, gout is well treatable and a combination of non-pharmacological and pharmacological treatments is recommended [3]. Urate-lowering therapy (ULT) should be considered and discussed with every patient after a first gout flare [3–5]. Yet, the management of gout in real-life is far from optimal. This has been attributed to patient, physician, and system factors [4, 6].

Adherence to prescribed ULT ranges from 20 to 70% and is considered to be among the poorest of all chronic conditions [7–9]. Patients’ barriers have been categorized into four areas: (1) limited gout knowledge; (2) few cues and feedback from direct environment and low frequency and quality of interactions with physicians; (3) negative attitudes towards and experiences with medication; and (4) failure to cope with practical barriers for long-term medication use [4, 10]. Patients' self-care behavior is a key determinant to modify these barriers of medication adherence [7, 11, 12]. Also, as part of quality of care, physicians are called upon to promote patient-centered care. This encompasses care that is responsive to the needs and preferences of patients [13]. Yet, self-management interventions can be time consuming in clinical setting. eHealth offers the opportunity to enhance self-management, while remaining efficient in a clinical healthcare setting. eHealth interventions have shown to be easy to use, have fewer availability restrictions, and temper pressure on healthcare systems [14–16]. Moreover, computer-tailored technology allows patients to receive highly tailored and personalized feedback about their personal situations and advices on how to improve where needed. Eight eHealth programs were launched to enhance gout self-management in general [17–19]. Yet, none of these focused on ULT adherence behavior. Similarly, interventions to improve adherence to ULT are limited and none of them addressed self-care behavior, a key determinant of adherence [7, 17, 20].

The Integrated Change (I-Change) model consist of an assessment of the individual’s current behavior and motivation regarding a desired health behavior, and integrates the answers given during an online assessment into personalized advice and feedback generated by unique algorithms [21, 22]*.* Computer-tailored support tools based on the I-Change model have proven to be (cost)-effective in changing various complex health-related behaviors and their determinants, including: smoking cessation [23], reducing alcohol consumption [24], reducing fat nutrition intake [25], increasing physical activity [26], and improving type 2 diabetes mellitus (T2DM) medication adherence [16].

Despite the growing popularity of computer-tailored support tools and their proven efficacy, patients may still experience difficulties with the user interface of the support tool and may therefore discontinue program use [27, 28]. Usability studies enable developers to discover potential difficulties with the support tool and to explore engagement and users’ experiences. Perceived usability has been demonstrated to be an important determinant of an individual’s intention to implement behavioral change, but also of actual use of the proposed intervention in clinical practice [29–31].

The aim of this study is to develop and assess usability of a web-based patient-tailored I-Change tool to support ULT adherence among gout patients in a clinical setting.

## Implementation

### Scope

The I-Change Gout tool aims to be used in clinical care to support ULT adherence among gout patients who are using ULT for at least 1 month, in whom ULT is adjusted, or in whom medication adherence was (suspected to be) suboptimal. While the focus is on ULT adherence, the I-Change Gout aimed explicitly to address lifestyle as an integral part of management.

### Development I-Change Gout tool

**Fig. 1:**
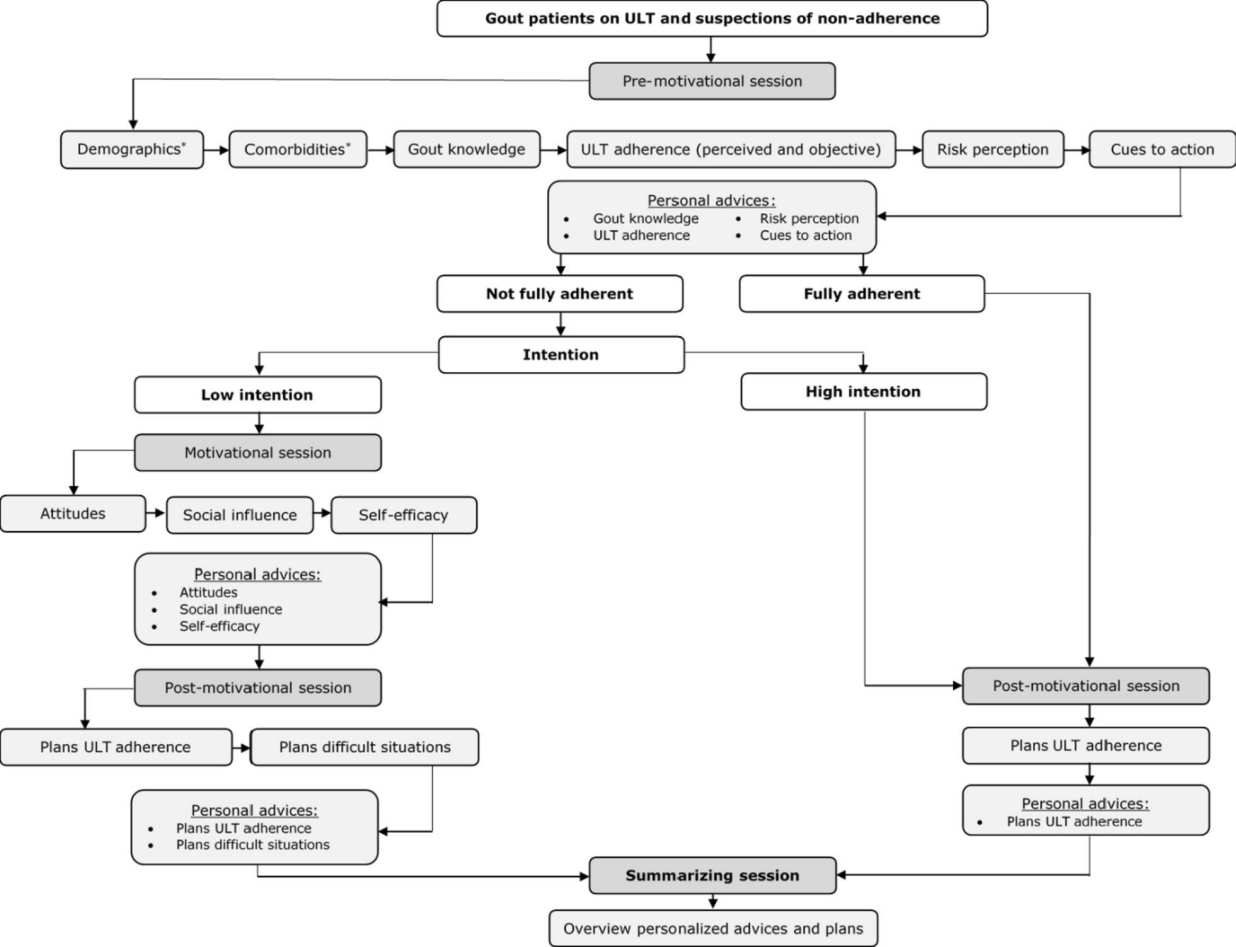
Flowchart of the I-Change Gout tool to support urate-lowering therapy adherence. *ULT* urate-lowering therapy. *Demographics and comorbidities were not used to provide patient-tailored advices. Yet, demographic information on marital status was used in the algorithm of social influence

The I-Change model combines various socio-cognitive theories and assumes that behavioral change is a result of becoming aware of the necessity to adjust one’s own behavior by integrating three phases: pre-motivational, motivational, and post-motivational [21, 32, 33]. The I-Change Gout tool assesses these 3 phases of behavioral change along three consecutive sessions and integrates pre-motivational factors (6 factors), motivational factors (3 factors), and post-motivational factors (2 factors) (Fig. 1) [34]. Details on the factors are described in Table 1. After each session, patients receive tailored feedback in the form of animated videos and text messages individualized to their answers on the questions (Additional file 1: table S1). As shown in Fig. 1, patients can navigate through the system following two trajectories, depending on (a) stated and revealed health behavior, and (b) the intention to adjust behavior. If the patient is considered to have a fully desirable health behavior following session 1, the patient is directed immediately to session 3. If the patient has suboptimal health behavior following session 1, the intention to adapt the behavior is assessed. In case of a low intention (i.e. not motivated to adapt), the patient is directed to session 2 (motivational session). A patient with a high intention (i.e. motivated to adapt), is immediately directed to session 3 (post-motivational session). All patients follow session 3, in which a patient is prompted to set specific goals and plans to adjust health behavior. The I-Change Gout tool ends by providing the patient an overview of the received advices and the plans for action made.

**Table 1:** Description of the sessions and the different factors assessed along the I-Change Gout tool, and the source of the questions

Sessions	I-Change factors	Content specific for the I-Change Gout tool	Source
I: Pre-motivational		To improve person’s awareness of the importance of ULT and their personal behavior towards ULT adherence	
	Demographics	Socio-economic background (e.g. age, gender, educational level, marital status, and work situation)	Adapted from previous effective I-Change tool and input from experts
	Comorbidities	Common diagnosed comorbidities influencing the management and control of gout	Rheumatic Disease Comorbidity Index questionnaire adapted for the purpose of the tool [59]
	Gout knowledge	The understanding of factual information regarding gout related to the pathogenesis, treatment of acute attacks and also management of chronic gout	Gout Knowledge Questionnaire adapted for purpose of the tool [36]
	Perceived ULT adherence	Person’s perception about his or her own ULT adherence behavior	Previous effective I-Change tools
	Objective ULT adherence	The degree to which the person’s ULT adherence behavior corresponds with recommended ULT use from a health care provider	ProMAS questionnaire adapted for ULT use [46]
	Risk perception	The perceived risk of gout flares or other gout problems as a result of non-adherence to ULT	Adapted from previous effective I-Change tool and input from experts
	Cues to action	Hints or signals a person is perceiving within his/her environment (external) or within him/herselff (internal) that trigger an action linked to the ULT adherence behavior	Adapted from previous effective I-Change tool and input from experts
	Intention	A person’s motivation in the sense of his or her conscious plan or decision to improve the ULT adherence behavior. The intention to adapt behavior detemines the navigation of the sessions. Decisive to move first to session II or immediately to session III	As in previous effective I-Change tool
II: Motivational		To improve motivation to take action regarding their ULT adherence behavior	
	Attitudes	A person’s overall evaluative opinion about their ULT adherence behavior as a result of the perceived advantages and disadvantages of the ULT adherence for this person	Adapted from previous effective I-Change tool and literature
	Social influence	The processes whereby person’s thougths, feelings, and actions about ULT adherence are directly or indirectly influenced by others	Adapted from previous effective I-Change tool
	Self-efficacy	The level of one's own belief to successfully carry out the desired ULT adherence behavior in certain difficult situations	Adapted from previous effective I-Change tool and input from experts
III: Post-motivational		To support patients in translating intentions into pre-formulated actions and coping plans to promote desired behavior	
	Plans ULT adherence	The process of choosing and planning specific actions and plans that may help to successfully adopt and maintain the ULT adherence behavior	Adapted from previous effective I-Change tool
	Plans difficult situations	The types of plans needed to maintain a behavioral change attempt and can contribute in a person’s pursuit to cope and overcome obstacles and difficulties by anticipating how to address these obstacles and difficult situation	Adapted from previous effective I-Change tool

*ULT* urate-lowering therapy

To develop the I-Change Gout tool a project group was assembled consisting of five gout experts, three health services researchers, and one health behavior expert. The perspective of the gout patients (the potential end-users) was explicitly included in the development by a qualitative study that was performed in preparation of the current study [35]. Three meetings of 2 h were scheduled to decide on the content of each of the three sessions. The project group had to agree on: (a) the questions and questionnaires required to assess various factors in the three sessions, (b) the cut-off points of scores on questions and questionnaires decisive for personalized feedback and navigation through the system, and (c) the content of the personalized feedback. A researcher (RtK) prepared the content of the meetings based on the preceding qualitative study among gout patients [35], literature on ULT adherence [4, 7, 8, 36], and individual contact with experts. Preparatory materials were sent two weeks before each meetings to the project group. An existing successful I-Change tool to support medication adherence in T2DM was used as basis [16].

### Usability study

A cross-sectional mixed methods design was used to evaluate usability among gout patients and healthcare professionals. A cognitive debriefing study using a think aloud approach was performed among patients and a series of validated usability questionnaires was completed by patients and healthcare professionals. Cognitive debriefing by individual think aloud sessions is an accepted approach to evaluate usability among patients and healthcare professionals [37]. The ethical committee of Maastricht University Medical Center (METC 2019-1040) approved this study and all participants provided written informed consent.

### Participants and procedure

Patients were eligible if they were ≥ 18 years, had sufficient knowledge of the Dutch language, and were currently using ULT. Patients were recruited from one regional non-university and one university hospital with a regional function. Patients were purposefully sampled to ensure representation of relevant age categories (≤ 50, 51–70, 71–85 and ≥ 86 years), gender (20% female), education levels (low, intermediate, and high), and disease duration (range 0–10 years). Healthcare professionals were recruited in hospitals and general practitioner practices in the south of the Netherlands and eligible if they were involved in gout management, but not in the development of the I-Change Gout tool. A sample size of ± 20 patients and ± 6 healthcare professionals was considered, as it is widely assumed that 5 participants suffice for usability testing and with 20 users 95% of the problems are captured [38].

### Think aloud

The think aloud study was conducted at the outpatient clinics in the presence of a researcher and was audio recorded. Patients were invited to log on and follow the instructions presented in the program, and complete the full program. Patients were asked to verbalize their thoughts and opinions while using the I-Change Gout tool, to assess patients reasoning and source of their problems. The researcher emphasized that the intention was to evaluate the program and not the participants’ behavior in order to encourage the participants to talk freely and express their positive and negative experiences.

### Usability questionnaire

After completing the full program all patients and healthcare professionals were invited to rate the usability of the I-Change Gout tool by completing a series of five validated questionnaires assessing four domains of usability:

- S*ystem usability* comprises four subdomains of the System Usability Scale [39, 40] evaluating: *strengths* (4 items; e.g. “I thought the program was easy to use”); *weaknesses* (3 items; e.g. “I thought there was too much inconsistency in the program”); *barriers* (2 items; e.g. “I needed to learn a lot before I could get going with the program”); and *intention* (2 items; “I think that I would like to use this system frequently” and “I think I would recommend others to use the system”).
- *Engagement* consists of 3 items (e.g. “The program made me curious”), adapted from of the Digital Behavior Change Interventions Engagement Scale [41, 42].
- *User experience* addresses 5 subdomains evaluating: *effectiveness* (3 items; e.g. “The program gives important information on the benefits of using ULT”); *trustworthiness* (3 items; e.g. “The program is trustworthy”); *enjoyment* (3 items; e.g. “I found the use of this program enjoyable”); *active trust* (3 items; e.g. “I know now how to use my ULT drugs better”); and *design aesthetics* (3 items; e.g. “I think the design of the program is attractive”) [43]. The original questionnaire also employs the subdomain “efficiency”, which measures the ease of searching and accessing information [43]. As searching information was not applicable, instead, the subdomain *design aesthetics* was added [44].
- *Program clarity* is measured by 11 items (e.g. “To what extent do you think this part of the gout tool [e.g. knowledge] is clear to use?”)

All questionnaire items are scored on a 5-point Likert scale. For the first three (sub)-domains, the anchors ranges from 1 = “I totally disagree” to 5 = “I totally agree”. For the domain *program clarity* the anchors ranges from 1 = ”very unclear” to 5 = ”very clear”. The total score for (sub)-domains is calculated as the average of the items, except for *intention* where the individual items are considered separately.

Finally, *program end score* was measured by asking participants to grade the gout tool on a numeric rating scale ranging from 1 = “very bad” to 10 = “very good”.

All questionnaires were rephrased to fit the perspective of the healthcare professionals. Following the questionnaires, participants were prompted to further clarify some response by written feedback in a single textbox.

### Analyses

Results of questions of the usability questionnaire were analyzed using descriptive statistics (e.g. mean and standard deviation) using IBM SPSS, version 25.0 (IBM Corp). Feedback in the textbox was linked by the researcher (RtK) to (sub)-domains of the usability questionnaire. The think aloud sessions were transcribed verbatim, anonymized, and analyzed (categorized in themes for each of the I-Change (sub)-sections) by a junior researcher (RtK) trained in qualitative research, and were checked by a senior researcher (AB). The Standards for Reporting Qualitative Research (SRQR) guided the transparency of all aspects of this qualitative research [45]. Textual remarks on written and spoken text or feedback on animated videos were collected per page of the I-Change Gout tool. All citations of patients were linked to the different (sub)-domains of the usability questionnaire. Expressed thoughts and opinions were used as input to improve the I-Change Gout tool if considered relevant after discussion within the project group. The revised I-Change Gout tool was tested among the following participants.

## Results

### Development I-Change Gout tool

The project group decided on the specific content of the I-Change Gout tool during the three meticulously prepared meetings. Details of the content and source feeding the content can be found in Table 1 and in the Additional file 1: data S1 and table S1. ULT adherence behavior is a key determinant for navigation through the system. In the I-Change Gout tool, patients are classified as optimal (opposed to suboptimal) adherence by combining questions on perceived and objective adherence. The Probabilistic Medication Adherence Scale (ProMAS) was chosen to assess objective adherence, as this instruments provides insight in the broad spectrum of (non)-adherence behavior [46]. To classify a person as optimal adherent, a strict cut-off point was chosen (fully self-perceived adherence combined with a 100% score on the ProMAS). Although a score ≥ 80% on the ProMAS is the formal threshold for acceptable adherence, the project group considered there would be room for improvement and potential value for patients to follow the I-Change Gout tool [46]. Patients with a suboptimal adherence and a low intention to adjust behavior navigate through all 3 sessions. Patients with a suboptimal adherence and high intention navigate after session one immediately to session three (as they can skip the motivational setting) (Fig. 1). Overall, the three sessions of the I-Change Gout tool consisted of 80 questions, 66 tailored textual feedback messages, and 40 tailored animated videos. Additionally, all patients have the opportunity to view evidence-based lifestyle advices [47]. After finalizing the content, the design (e.g. avatar) was discussed, and textual information was adapted to health literacy basic reading levels.

### Usability study

Twenty gout patients and seven healthcare professionals participated in the usability study. Patients were 69.6 ± 14.7 years old, 85% (17/20) were male, had a mean disease duration of 8.3 ± 9.7 years (median: 5.0), with education levels ranging from high (n = 5; 25%), intermediate (n = 7; 35%), to low (n = 8; 40%). Healthcare professionals were 38.0 ± 13.7 years old, 43% (3/7) were male, working experience was 10.4 ± 11.8 years (median: 5.0), and professional background ranged from general practitioner (n = 2; 29%), rheumatologist (n = 2; 29%), occupational physician (n = 1; 14%), to a physician assistant (n = 2; 29%).

### Usability questionnaires

Table 2 presents the scores of the patients and healthcare professionals on the usability domains. The *program end score* rating was on average 8.4 ± 0.9 (range 6–10) for patients and 7.7 ± 1.0 (range 6–9) for healthcare professionals. Intention to use the system in the future and recommend it to others was high among patients (average: 4.4 ± 0.6 and 4.6 ± 0.6, respectively) and healthcare professionals (average: 4.0 ± 0.0 and 4.0 ± 0.6, respectively).

**Table 2:** Usability according to patients and healthcare professionals

Domains	Subdomains	Patients (n = 20)	Healthcare professionals (n = 7)
System usability^a^	Strengths	4.4 (0.6)	4.3 (0.5)
	Weaknesses	1.3 (0.6)	1.8 (0.5)
	Barriers	1.6 (1.0)	1.4 (0.5)
	Intention		
	*to use the system*	4.4 (0.6)	4.0 (0.0)
	*to recommend the system*	4.6 (0.6)	4.0 (0.6)
Engagement^a^	Engagement	4.2 (0.7)	3.4 (0.3)
User experience^a^	Effectiveness	4.0 (0.8)	3.9 (0.8)
	Trustworthiness	4.5 (0.5)	4.4 (0.5)
	Enjoyment	4.3 (0.6)	3.6 (0.4)
	Active trust	4.1 (0.8)	4.1 (0.4)
	Design aesthetics	4.4 (0.7)	4.0 (0.1)
Program clarity^b^	Program clarity	4.1 (0.4)	3.9 (0.4)
Program end score^c^	Program end score	8.4 (0.9)	7.7 (1.0)

^a^1 = strongly disagree, 2 = disagree, 3 = neutral, 4 = agree, 5 = strongly agree

^b^1 = very unclear, 2 = unclear, 3 = neutral, 4 = clear, 5 = very clear

^c^1 = very bad to 10 = very good

Overall, no striking low scores were observed. Among healthcare professionals, average scores were more frequently below four (Table 2). The lowest score by healthcare professionals were found for engagement (3.4 ± 0.3) and enjoyment (3.6 ± 0.4).

In the open questions, healthcare professionals appreciated the interactive and personalized provision of valuable information of gout management and ULT adherence:

Short, clear and good supporting animations, that is the strength of this intervention (HP3).

Notwithstanding, one healthcare professional raised worries on the ability of patients to become an actor of their own health behavior:

I am afraid that unmotivated gout patients may not be motivated by this [program] either, it will not achieve its goal and it will only be developed for the small group that is already serious about his/her disease (HP5).

Further, professionals questioned the skills of the elderly gout patients, and their health literacy. In the open questions, some patients mentioned technical issues with regard to the use of the I-Change Gout tool.

### Think aloud

Patients appreciated the information and application of the three sessions, and described them as positive, useful, and clear. The variation and interaction between video- and text-based advices was experienced positively (Table 3). Furthermore, patients appreciated videos and text length, stating it was short and informative:

The written material describes gout well and focuses on the importance of regularly taking ULT (PT 13).

**Table 3:** Citations of patients during the think aloud study related to the different subdomains of the usability questionnaires

Domains	Subdomains	Citations
System usability	Strengths	Informative and easy to use—it is patient friendly
	Weaknesses	The medication questionnaire has often repetitive questions
	Barriers	I need support to use a digital program, not using a computer in daily life
	Intention	I definitely will use the program if it is available for me
Engagement	Engagement	I am going to implement these plans and I am very curious about the program
User experience	Effectiveness	With the information I have just heard, I think I should use my tablets daily, even if I have no gout complaints. I will start using my pill box again
	Trustworthiness	The written material described gout well and focused on the importance of regularly taking ULT
	Enjoyment	I liked the lay-out of the program, and it was interesting to go through the program
	Active trust	I have made plans to improve my tablet use, and my advices were clear
	Design aesthetics	Short, clearly and good supporting video’s
Program clarity	Program clarity	The entire program is clear and I have no trouble filling in the questions

Patients indicated that the I-Change Gout tool was effective and gave important information on the benefits and importance of ULT. The program helped to consider taking ULT as prescribed:

With the information I have just heard, I understand I should use my tablets daily, even if I have no gout complaints. I will start using my pill box again (PT 1).

This is the first time I hear that allopurinol is involved in the treatment of gout (PT7).

Improvements were suggested in language use of written text such as shortening or rephrasing some feedback messages, and replace words. In addition, patients suggested explaining the system navigation more explicitly at different parts of the three sessions to improve the effective use. Furthermore, categories on education and work situations were revealed to be missing on the initial page asking patients to tell about their socio-economic background. Finally, one additional ULT adherence plan was suggested (see text PT8). These smaller changes were immediately implemented in the I-Change Gout tool.

Add ‘using a reminder app on your smartphone’ as specific plan; this slightly differs from an alarm as an alarm is easy to click away. I will then still forget the ULT (PT8).

Four patients had some critical remarks regarding the objective ULT adherence questionnaire (ProMAS) in session 1. One patient stated for example:

I don't feel taken seriously by this questionnaire, too often it boils down to the same thing and this annoys me (PT2).

Yet, another patient indicated to clearly recognize the added value of the ProMAS questionnaire:

There are often repetitive questions, yet there is a difference in dimensions and this makes you really think about your use of tablets (PT8).

To avoid feedback messages for each individual item of the ProMAS (n = 15), feedback was clustered by items addressing a similar construct after discussion within the project group.

For a minority of patients that were adherent to ULT, according to the ProMAS questionnaire during completing the program, the program had less added value:

I already have a lot of gout knowledge, and use my tablets daily, so the system may be less effective for me. However, the program would certainly be valuable for patients who are new with gout or do not have the gout knowledge like me (PT6).

On a same line, adherent patients did not entirely recognize the benefit of making plans to stay adherent as they already made specific plans for ULT use. However, for patients with ULT adherence issues, reminders to take ULT on a daily basis were frequently mentioned as a useful coping plan. Overall, making coping plans and having a daily routine was reported by almost every patient as necessary for daily tablet use:

Place the tablets in a fixed place, and be very precise in this. This is also necessary to take them properly (PT19).

## Discussion

This study describes the development and usability of web-based patient-tailored tool to support adherence to ULT in gout patients in a clinical setting. Both patients and healthcare professionals reported a high intention to use and/or recommend the tool to others. No major problematic issues were identified across the domains of usability questionnaires, yet healthcare professionals raised some worries about engagement of elderly patients, those that have poor digital literacy, and those intrinsically unmotivated. The specific points for further improvements (e.g. repetition of questions, technical issues, and readability of text) revealed by participants were immediately adjusted following each interview until the current final version of the I-Change Gout tool.

Lack of knowledge has been identified as an important determinant of ULT adherence [4]. Patients indicated that the current I-Change Gout tool was effective as it addressed knowledge gaps and inadequate risk perceptions effectively by actively improving patients’ knowledge and risk perception, and rectified several misconceptions with tailored animated videos and text messages.

In addition to knowledge, patients’ motivation is key in changing the self-care behavior. The I-Change Gout tool was developed with the intention to improve patients’ motivation and support the complex ULT adherence behavior within three sessions. The motivational session ensured that patients with suboptimal adherence and a low intention became aware of the added value of ULT adherence, by addressing attitudes (pros and cons of ULT), social influence (support and norm to use ULT), and self-efficacy (action plans on ULT use) effectively to promote desired behavior regarding ULT use. Notwithstanding, healthcare professionals doubted whether the I-Change Gout tool would truly reach the desired medication adherence behavior in less motivated patients. To gain insight into the magnitude and potential solutions for this problem, more in depth qualitative and quantitative evaluation of the I-Change Gout tool in less motivated patients will be required. For patients that are less motivated, direct support and encouragement to follow the I-Change Gout tool by healthcare professionals, who should be aware of their role as social influencer, may still be needed.

Furthermore, in the post-motivational session patients’ clearly revealed that coping plans and a daily routine are valuable for ULT adherence. Literature supports the fact that coping plans were associated with ULT adherence among gout patients [48, 49]. Although adherent patients did not entirely recognize the benefit of making plans to stay adherent, it remains important to make coping and action plans in the post-motivational session to remain adherent.

The current I-Change Gout tool is the first web-based patient-tailored tool that specifically addressed ULT adherence in gout patients based on various theories that influence health behavior through self-management. The I-Change Gout tool was designed to complement usual care at the first visit following implementation of ULT, and addresses desired lifestyle behavior in addition to importance of adherence to ULT. In the current study, patients highlighted that voiced animated video-based advices were preferred over long pieces of text. The videos were rated as informative, of adequate length, and sufficiently personalized to foster good acceptance, engagement, and intention to use the program. That video tailoring can be effective and may be preferred over text tailoring was confirmed by existing studies [50, 51].

A randomized controlled trial will be required to assess the efficacy and (cost-) effectiveness of the I-Change Gout tool in daily practice. Such a trial should also clarify what the uptake of the tool is and in which subgroups of gout patients (at risk) the tool will be effective (i.e. most relevant target group). Patients with lower computer or health literacy skills may be less likely to use the tool [52] and direct support by healthcare professionals or social support may still be needed for those patients. The Netherlands ranks among the European top in digital skills, yet health literacy is still considered problematic or inadequate in respectively 26.9% and 1.8% of persons in the general population [53]. Patients with gout have shown to score even lower in various domains of health literacy than patients with other rheumatic diseases [54]. The I-Change Gout tool specifically tried to reduce reading burden and increased accessibility to low-literacy patients by using short sentences and plain language, multimedia formats including pictures and videos, and dropdown options to reduce reading time and improving comprehension [55]. Of note, when providing patient-centered care, it is equally important identify patients that prefer individual learning opposed to those in which person-to-person contact is more effective.

A review of 18 interventions that aimed to improve medication adherence of gout patients [20], found that nurse-led interventions with patient education is the most promising in achieving improved adherence compared to usual care [56, 57]. The authors discussed that none of the interventions addressed to develop self-care behavior (e.g. action plans), despite evidence of its relevance in medication taking behavior. Potentially, the I-Change Gout tool could be efficacious and even more cost-effective compared to nurse-led approaches [16]. An interesting review of mobile applications to improve adherence through self-management of gout patients build upon effectiveness of regular feedback on disease control [17]. They found six apps that educate patients and help them to monitor their sUA. One of these fulfilled predefined quality criteria [17]. As it is known that informed decision (as I-Change) improves uptake and short-term adherence to medications, such an app could be considered as part of the action plans to ensure long-term adherence.

Although several challenges of the tool have been mentioned above, one limitation should be specifically discussed. The ProMAS was chosen to estimate objective medication taking behavior. The questionnaire yielded some negative feedback (e.g. repeating questions). Adaptations (e.g. clustered feedback messages on the answers from repeating questions of the ProMAS) and more clarification of its reasoning (e.g. to make the tool personal tailored) were implemented and should potentially lead to better enjoyment. Additionally, the ProMAS was not validated as objective adherence measurement among gout patients. However, the ProMAS is a better way to quantify adherence behavior, as it assesses a range of medication taking dispositions with varying difficulty levels using a Rasch model approach [46]. The ProMAS yielded insight in a broader spectrum of adherence behaviors compared to the most frequently widely used Medication Adherence Report Scale (MARS) [58]. Furthermore, the ProMAS was tested among patients receiving medication for chronic conditions. Although it may be unlikely that the validity will differ between various chronic diseases, specific research is needed to be able to answer this question. Overall, based on all the methodological considerations, we feel the ProMAS was the best fit for our purpose as objective medication adherence measurement Lastly, for the purpose of the I-Change Gout tool, we had to adapt several questionnaires from the literature in order to comply as closely as possible with the I-Change model factors, yet the tool can be easily adopted when better/validated instruments are published. The current study demonstrated that a systematic development process based on evidence from literature, views of experts, and perspectives of gout patients is important. Although the synthesis and interpretation of the findings of the cognitive debriefing and the open answers of the usability questionnaire were intensively discussed within the project group, coding and analysis of the think aloud sessions was conducted by only one researcher. As the feedback was quite straightforward, and the themes syntheses of the verbatim transcripts and themed summaries were checked by a senior researcher, it is unlikely that the interpretation might be biased. A transparent description of the development is a first and essential step towards understanding effectiveness of any support tool. Other researchers or tool developers can use the methodological development process of the I-Change Gout tool as guidance in their own development process.

## Conclusion

This study provides initial support for the usability by patients and healthcare professionals of an I-Change gout tool to support ULT adherence behavior. Further studies need to be conducted to assess its efficacy and (cost-) effectiveness in daily practice.

## Availability and requirements

- Project name: The I-Change Gout Tool
- Project home page: https://tailorbuilder.ose.nl/cgi-bin/runtime.pl?a=NAM2V8B960PFJ44B6859A16Y74HGFV
- Operating system: TailorBuilder
- Programming language: Java
- License: OverNite Software Europe BV
- Any restrictions to use by non-academics: licence needed, and currently only available as backhand program. Not entirely live yet.

## Supplementary Information

**Additional file 1.** Content I-Change Gout tool.

## Data Availability

The raw data (e.g. results of usability questionnaire or think aloud sessions) that support the findings of this study are available on reasonable request from the corresponding author (RtK).

## Abbreviations

ULT: Urate-lowering therapy
I-Change: Integrated Change
T2DM: Type 2 diabetes mellitus
ProMAS: Probabilistic Medication Adherence Scale
