# Supplementary material for: Development and usability of a web-based patient-tailored tool to support adherence to urate-lowering therapy in gout
Source: BMC Med Inform Decis Mak. 2022 Apr 7;22:95. doi: 10.1186/s12911-022-01833-6 (PMC8991610; doi:10.1186/s12911-022-01833-6)
Supplement: Supplementary file 1 — Additional file 1. Content I-Change Gout tool. [file 12911_2022_1833_MOESM1_ESM.docx]

**Supplementary Data S1**

**Content I-Change Gout tool**

**I: Pre-motivational session**

*Demographics*

Patients were asked to indicate their gender (1 = Male, 2 = Female, 3 = Other), age, height, weight, highest completed educational level, marital status, and current work situation. Educational level was categorized into 1 = No education, 2 = Primary education, 3 = Lower vocational school, 4 = Secondary education, 5 = Secondary vocational education, 6 = Secondary general education, 7 = Higher education, 8 = University education, and 9 = Otherwise. Marital status was categorized into 1 = Married/registered partnership, 2 = In a relationship (not living together), 3 = In a relationship (living together), 4 = Single/divorced, 5 = Widow, and 6 = Otherwise. The current working situation was categorized into 1 = Paid work, 2 = Housewife/husband, 3 = Retired, 4 = Study, 5 = Volunteer work, 6 = Unemployed/jobseeker, 7 = Disabled/incapacitated for work, 8 = Self-employed, and 9 = Otherwise.

*Comorbidities*

Patients were asked if they were ‘Never treated for’, ‘Treated in the past’ or ‘Treated currently’ for comorbidities. Comorbidities were categorized into 1 = Lung diseases, 2 = Heart attack/myocardial infarction, 3 = Other heart problems, 4 = Stroke, 5 = Hypertension, 6 = Fracture of the back, hip or leg, 7 = Depression, 8 = Diabetes, 9 = Peptic ulcer or other stomach problems, and 10 = A form of cancer.

*Gout knowledge*

Gout knowledge was measured by an index of ten items. Patients were presented with ten statements such as ‘Gout is caused by too much uric acid in my blood’, and were asked to answer the statements with ‘True’, ‘False’, or ‘I don’t know’.

*ULT adherence*

Urate-lowering therapy adherence was assessed by two items. Firstly, patients were asked to what extent they agreed with the statement ‘I am currently taking my urate lowering tablets fully as agreed with my healthcare provider’. The answering option ranged from 1 = ‘Absolutely disagree’ to 5 = ‘Absolutely agree’. Secondly, urate-lowering therapy adherence was measured with the ProMAS questionnaire. The ProMAS consisted originally of eighteen items that assess behaviors, e.g. forgetting, stopping, changing dosages, or taking medication too late. Three questions about “the moment of intake daily” were deleted as they were not important for gout adherence. To be able to compare changes over time, we further added a time period of one month to every single items of the ProMAS to assess adherence of the past month. The answering options of the fifteen items were ‘True’ or ‘False’.

*Risk perception*

Risk perception was measured by two items. Patients were asked how they think about the risk of gout if they not taking urate lowering tablets with answering options from 1 = ‘Absolutely disagree’ to 5 = ‘Absolutely agree’.

*Cues to action*

Two items were used to ask patients about cues to use medication as prescribed. Cues included for example ‘Experienced gout attacks within the past three months’. Answering options ranged from 1 = ‘No, definitely not’ to 5 = ‘Yes, definitely’.

*Intention*

Intention was measured by one item. Patients were asked whether they were planning to use the urate lowering tablets daily within the next month. The answering option ranged from 1 = ‘No, definitely not’ to 5 = ‘Yes, definitely’.

**II: Motivational session**

*Attitudes*

Attitudes were assessed by 12 items. Patients were asked to indicate to what extent they agreed with statements following the stem ‘If I am using the urate lowering tablets every day… ’. Six items measured cons (negative attitudes) such as ‘It costs a lot of money’ or ‘I worry about side effects’, another six items concerned pros (positive attitudes) such as ‘I have less gout flares’ or ‘I can do my daily activities’. Answering options were recoded from 1 = ‘Absolutely disagree’ to 5 = ‘Absolutely agree’.

*Social influences*

Social influence was measured by six items and included social influence of the partner, family members, and friends. Three items concerned norms, asking patients to answer statements such as ‘My family members think that I should use my urate lowering tablets daily’ with the answering options from 1 = ‘Absolutely disagree’ to 5 = ‘Absolutely agree’. The other three items concerned support, asking patients to answer statements such as ‘My partner stimulates me to use my tablets daily’. Answering options ranged from 1 = ‘Totally disagree’ to 5 = ‘Totally agree’.

*Self-efficacy*

Self-efficacy was assessed by ten items following the stem ‘I find it difficult/easy to use my urate lowering tablets daily if…’. Items included the following situations: 1 = I’m busy, 2 = I’m sick, 3 = I have an unstructured day, 4 = I’m at a party, 5 = I’m gloomy, 6 = I’m eating out, 7 = I’m on vacation, 8 = I have a gout flare, 9 = I have visitors, 10 = I’m stressed. Answering options ranged from 1 = ‘Very difficult’ to 5 = ‘Very easy’.

**III: Post-motivational session**

*Plans ULT adherence*

Patients were prompted to set specific goals for their urate-lowering therapy adherence behavior. Action planning was assessed by eight pre-formulated options following the stem ‘I’m planning to…’. Options included for example, ‘Use a pill box’ or ‘Have my urate lowering tablets on a fixed place’. Patients are encouraged to set small, realistic and achievable plans and can make multiple plans.

*Plans difficult situations*

Coping plans were made for their difficult situations assessed within self-efficacy section. Patients were provided with ten pre-formulated options following the stem ‘If I want to use my tablets daily, but [specific difficult situation], then…’. Options included for example, ‘I take my tablets before I leave home’.

**Supplementary Table S1**

Table S1: Description of examples of feedback messages of the sessions and the different factors assessed along the I-Change Gout tool

| **Sessions** | **I-Change factors** | **Examples of feeback messages** |
| --- | --- | --- |
| I: Pre-motivational | Gout knowledge | Dear patient, you already have a lot of knowledge about gout, but you can still learn some things about gout, such as whether gout can be cured, whether you should take ULT tablets long-term, and whether red meat lowers sUA? |
|  | ULT adherence | Dear patient, we have just asked you some questions about your ULT intake. You think that you are taking your ULT tablets as agreed with your healthcare provider and this is true. Great that you are aware of this behavior. Yet, there are still a few small points that you can improve. |
|  | Risk perception | Dear patient, you are fully aware of the risk of not taking your ULT tablets. It is very good that you are aware of this risk perception. When you not taking your tablets, sUA rising starts again and gout flares may return. |
|  | Cues to action | Dear patient, you indicated that you did experience personal (internal and external) cues to take action to use the ULT tablets in the prescribed manner. Is this a reason to change you behavior of taking the tablets? In any case, it is important that you continue to take your tablets in the prescribed manner and on a daily basis |
|  | Intention | Dear patient, you do not want to improve your tablet use within a month. That is too bad. Your tablet use is currently insufficient, so you have the risk of having a (new) gout flare if you don't take the tablets in the prescribed way. In the remainder of the program we will try to make you even more aware of the benefits of using tablets. |
| II: Motivational | Attitudes | Dear patient, fortunately you see many benefits from the use of ULT tablets. Of course this also has many advantages. You also indicate that you see some disadvantages for the use of ULT tablets. The benefits outweigh the potential downsides, as using your ULT tablets as faithfully as possible will help you live with your gout. |
|  | Social influence | Dear patient, you experience no support from people close to you to take your ULT tablets. That is a shame, support can always help you improve your ULT adherence behavior. If you don't feel support, maybe you can ask people you know for help? |
|  | Self-efficacy | Dear patient, if you go out for dinner, if you are at a party, if you are busy or have an unstructured day, or if you may be stressed, you indicate that you find it more difficult to take your ULT tablets. How did that happen? And why are it precisely these situations? To make sure that you use your tablets in these situations, it helps to make a plan. We will tell you how to make a good plan later in this program. |
| III: Post-motivational | Plans ULT adherence | Dear patient, you have developed the following plans [plans are shown] that can help you achieve your goal of improving ULT adherence. Below your plans are additional tips that can help you to implement these plans successfully. |
|  | Plans difficult situations | Dear patient, you have identified difficult situations for yourself and come up with plans for them: plans that can help you deal with these difficult situations. Below you will find an overview of your difficult situation with associated plans. |

ULT= urate-lowering therapy, sUA= serum uric acid
